# A dichotomy of smokers in the Philippines following sin tax reform: Distinguishing potential quitters from those unlikely to quit

**DOI:** 10.1371/journal.pone.0275840

**Published:** 2022-10-13

**Authors:** Kent Jason Go Cheng, Miguel Antonio Garcia Estrada

**Affiliations:** 1 Social Science Department, Maxwell School of Citizenship and Public Affairs, Syracuse University, Syracuse, New York, United States of America; 2 Department of Public Administration and Policy, School of Public and International Affairs, University of Georgia, Athens, Georgia, United States of America; LSU Health Sciences Center New Orleans: Louisiana State University Health Sciences Center, UNITED STATES

## Abstract

The Philippine government significantly raised cigarette excise taxes in 2013, following passage of the landmark Sin Tax Reform Act of 2012. As a result, cigarette prices increased substantially. Given varying smokers’ responses to the price increase, we examined underlying typologies of Filipino smokers and assessed how these typologies determine smoking intensity. We used cross-sectional data from the 2015 wave of the Philippine Global Adult Tobacco Survey (N = 1,651). To uncover typologies, random effects latent class modelling was used on six individual smoker responses (attempting to stop, thinking about quitting, decreasing sticks smoked, switching to cheaper brands, buying in bulk, and asking from others). Bivariate and multivariate analyses were employed to uncover determinants of typologies and smoking intensity. We found two typologies based on smokers’ response. The first group, called “potential quitters” (62.62%), is composed of smokers who are more likely to consider quitting and decrease sticks smoked. The second group, called “unlikely to quit” (37.38%), have smokers who opt for price-minimization strategies like switching to cheaper brands, buying in bulk, or asking cigarettes from others. Potential quitters tend to be female, a student, and less nicotine dependent. They smoke up to three fewer sticks than those unlikely to quit, controlling for other factors. Nicotine dependence stood out as the most important predictor of being in the unlikely to quit group. The dominant role of nicotine dependence in determining a smoker’s typology points to the need for non-price based measures, such as those targeted towards highly-nicotine dependent smokers, to complement tax-induced price increases and comprehensively address the smoking problem.

## Introduction

In 2019, tobacco accounted for about 8.17 million deaths globally [1]. Smoking prevalence, which has been declining in past decades, remains significant at approximately 25.0% for men and 5.4% for women [2].

About one in five Filipinos is a smoke [3]–making the Philippines the country with the third highest smoking prevalence in Southeast Asia [4]. Although prevalence has been declining, the annual rate of decline barely changed since 1990 [2]. That average smoking intensity or the number of daily sticks smoked has increased from 18.5 in 1980 to 21.4 in 2012 –a figure higher compared to more than half of the 187 countries studied by Ng et al. [5]–is another cause for concern. This increase in smoking intensity that happened alongside the decline in prevalence lends some support to the “hardening hypothesis,” which posits that tobacco control measures mainly affect less dependent smokers, leaving behind “hardened” smokers who would never quit smoking [6, 7].

The Philippine government significantly increased cigarette excise taxes by passing the Sin Tax Reform Act of 2012 [8]. Since then, a series of tax increases followed (see Table 1 for a summary of excise tax reforms). By 2015, the average price per stick of cigarette was estimated at PhP 2.46 or about 5 US cents [9]. This is about 1.5% of the 2015 average daily individual income in the Philippines of PhP 166.25 (about USD 3.3). The amount is based on authors’ computation using the 2015 average household income [10] and an average household size of 4.4 [11]. There is a convincing body of evidence [12, 13] pointing to tax increases as the “single most effective intervention to reduce demand for tobacco” [14].

**Table 1:** Pre- and post- 2012 reform excise tax amounts in Philippine peso (PhP).

**Pre-Reform**					
Republic Act Number (law)	Year	Tier 1 (NRP < PhP 5)	Tier 2 (PhP 5.00 ≤ NRP ≤ PhP 6.50)	Tier 3 (PhP 6.50 ≤ NRP ≤ PhP 10.00)	Tier 4 (NRP > PhP 10.00)
9334 of 2004	2012	PhP 2.72	7.56	12.00	28.30
**Post-Reform**					
RA Number	Year	Tier 1 (NRP ≤ PhP 11.50)		Tier 2 (NRP > PhP 11.50)	
10351	2013	12.00		25.00	
	2014	17.00		27.00	
	2015	21.00		28.00	
	2016	25.00		29.00	
	2017	30.00			
10963	2018	33.75			
	2019	35.00			
11346	2020	45.00			
	2021	50.00			
	2022	55.00			
	2023	60.00			
	2024	Annual increases of 5%			

Notes: RA = Republic Act, NRP = Net Retail Price. Amount in 2018 (PhP 33.75) is the average of PhP32.50 (January-June 2018) and PhP35.00 (July-December 2018), pursuant to RA 10963.

See Cheng & Estrada [9] and Estrada [15] for the specific tax amounts pre-2012.

Implementation of the landmark 2012 law was followed by a noticeable decline in smoking prevalence–from 28.3% in 2009 to 23.8% in 2015. However, smoking intensity among daily smokers slightly increased from 10.6 sticks per day in 2009 to 11.0 in 2015, according to the Global Adult Tobacco Survey (GATS) data [3]. This suggests that price increases affect smoking behavior more through the prevention of smoking initiation than the reduction in smoking intensity. One recent study found that while a 10% increase in price would lead to 12.4% *reduction* in the probability of smoking participation, the same price increase may lead to a 1.4–6.8% *increase* in sticks smoked [9].

The evidence on which pathway–smoking participation or intensity–excise taxes influence smoking behavior is rather mixed and lacking in the Philippine context. Moreover, not much has been done on smoking cessation determinants in developing countries like the Philippines [12]. These raise the importance of research on varying smokers’ responses to price-based interventions to understand their impact. In this regard, we aim to examine the influence of excise tax increases by uncovering typologies based on smokers’ response to price changes. We also aim to understand the characteristics of smokers in each typology and how they relate to smoking intensity. Using information on how increases in cigarette prices affected individuals’ smoking decisions following the tax reform, we posit that (1) some individuals resort to drastic reduction in smoking either by considering cessation or decreasing sticks smoked, while (2) others find ways to maintain their smoking habits by switching to a cheaper brand, buying in bulk, or asking cigarettes from fellow smokers.

## Methods

### Data

We used data from the 2015 wave of the Philippine Global Adult Tobacco Survey (GATS), the global standard for systematically monitoring adult tobacco use and tracking key tobacco control indicators [3]. The GATS has a stratified multi-stage cluster sampling design that is representative of the country’s population aged 15+. Primary sampling units (PSUs) consisting of either *barangays* (i.e., the smallest administrative division in the Philippines) or combinations of small contiguous *barangays* within the same municipality were randomly selected. Within PSUs, enumeration areas were identified. Housing units within each enumeration area were determined. The target population was sampled from the 13,963 housing units which yielded 11,644 completed individual interviews.

Person-level response rate was 96.3%. Incompleteness, absence from home, refusal, and incapacity were among the reasons cited for non-response or non-inclusion of certain respondents. Most of these (2.2%) were due to absence from home. A slightly higher share of 2.3% were in rural areas while urban dwellers accounted for the remaining 1.4%.

Indonesia [16], Malaysia [17], Thailand [18], and Viet Nam [19] are the other countries in Southeast Asia that implemented the GATS for the period preceding 2015. All four countries ran the survey either in 2010 or 2011, and of the four, Indonesia recorded the highest adult smoking prevalence at 36.1% in 2011 [16–19]. Thailand had a prevalence of 26.9% for the same year while Vietnam and Malaysia had lower figures at 23.8% (2010) and 23.1% (2011), respectively [16–19]. In relative terms, the Philippines had the second highest prevalence figure at 28.3%, based on the 2009 results of the GATS [16–20]. Among the Southeast Asian countries, only the Philippines, Indonesia, and Vietnam ran subsequent rounds of the GATS following the period 2009–2011 [16–20].

Our study is concerned with current smokers who claimed to have been affected by the 2012 tax increase. Of the total sample, 2,793 or 24.0% were current smokers, 87.7% of which were male. Smokers were asked this question: “The tax on cigarettes in the Philippines has increased in January 2013, January 2014, and January 2015, resulting in higher prices of cigarettes. Have the increases in cigarette prices affected your smoking?” Our study was confined to the n = 1,688 smokers who answered affirmatively and excluded those who answered *no* (n = 856) and *don’t know / refused* (n = 38). Our analytic sample numbered 1,651 after listwise deletion.

The GATS was conducted by the Philippine Statistics Authority (PSA), in cooperation with the Philippine Department of Health. The survey was approved by the ethical boards in the country and the dataset, with de-identified respondents, is publicly-available. Hence, this study does not require a separate ethical review and approval.

### Dependent variables

There are two main dependent variables: (1) typologies of how Filipino smokers responded to sin tax reform and (2) cigarette demand as measured by daily sticks smoked. For the typologies, the 2015 GATS directly asked smokers who claimed to have been affected by the tax increase how they adapted to the price change through this question: “In which of the following ways have the increases in cigarette prices affected your smoking?” The choices were: (1) making an attempt to stop (referred to henceforth as *Stop*), (2) thinking about quitting smoking (*Thought*), (3) decreasing the number of sticks smoked per day (*Decrease*), (4) switching to a cheaper brand (*Switch*), (5) buying cigarettes in bulk/ reams (*Bulk*), and (6) asking for cigarettes from other smokers (*Ask*). We dichotomized these variables such that 1 = affirmative response and 0 = otherwise.

### Independent variables

We controlled for the following: age, sex (1 = female, 0 = male), education (no education or elementary undergraduate, elementary graduate, high school graduate, with college graduate or higher as reference), employment status (unemployed, student, not in the labor force, with employed as reference), wealth index (in tertiles, with highest tertile as reference), nicotine dependence, type of residence (1 = rural, 0 = urban), presence of at least one smoker in the household, and an index for exposure to secondhand smoke.

The wealth index was constructed using principal components analysis (PCA) on variables representing amenities the respondent owns (e.g., own electricity) [9, 21, 22]. Nicotine dependence refers to time to smoking the first cigarette after waking up [23]. Choices were categorized as: (1) within 5 minutes [i.e., the most nicotine dependent], (2) 6 to 30 minutes, (3) 31 minutes to an hour, (4) after an hour, and (5) not a daily smoker. The index of secondhand smoke exposure was based on the number of places (e.g., workplace) the respondents saw someone smoking in the past 30 days.

Cigarette price was determined by assigning each respondent the average self-reported cigarette price for the respective PSU or type of residence when PSU is not available to avoid the endogeneity problem (see [24]).

### Statistical and sensitivity analyses

To determine the typologies of adaptations to price increases, we subjected *Stop*, *Thought*, *Decrease*, *Switch*, *Bulk*, and *Ask* to latent class modelling (LCM)–a technique that clusters observed variables into latent or unobserved categories [25, 26], in which an observation has a certain probability of being a member of a class. The standard LCM assumes that observed variables are independent of each other [26, 27] but this assumption is often unjustified due to correlated response errors and omitted variables [28, 29]. Example, decreasing smoking intensity and smoking cessation may be interdependent as many people opt for the reduction-to-quit instead of *cold turkey* [30]. Hence, we implemented the random effects (RE) version of LCM using the *randomLCA* R package [31] because it does not need observed variables to be independent of each other [25, 32]. Here, the probabilities of class membership are first transformed to either probit or logit scales, a normally distributed random effect will be added to each subject, and then the scales with the random effect would be transformed back to probabilities [31].

To help us decide on the number of typologies that fit the data best, we used Bayesian Information Criterion (BIC) (lower BIC, better model fit) and we compared actual versus fitted values. Multivariate logistic regression was then used to determine the respondent-level variables that predict class membership while robust ordinary least squares (OLS) regression was utilized to know whether class membership, controlling for other factors, was associated with smoking intensity. Smoking intensity was presented as both the daily number of sticks smoked and its log-transformed version as a sensitivity check.

## Results

Our sample has a mean age of 41 years, composed mostly of males (91%), high school graduates (42%), employed individuals (87%), those who smoked within 6 to 30 minutes after waking (30%), rural residents (65%), and has at least one smoker in the household (98%). Our sample smoked about 9 sticks per day and paid about PhP 3 per stick or about 6 cents USD (Table 2).

**Table 2:** Statistics by typology.

Variables	Total (N = 1,651)	Classes from the RE LCM with Logit Scale		Bivariate analysis
		“Unlikely to Quit” (N = 624)	“Potential Quitters” (N = 1,027)	
Price in PSU/ type of residence	Mean = 2.64; SD = 4.33	Mean = 2.54; SD = 2.94	Mean = 2.71; SD = 4.99	t = 0.78
Sticks smoked per day	Mean = 9.24; SD = 7.99	Mean = 11.28; SD = 8.65	Mean = 8.00; SD = 7.29	t = -8.26***
Age	Mean = 41.07; SD = 14.34	Mean = 40.99; SD = 14.37	Mean = 41.12; SD = 14.33	t = 0.19
Female	152 (9.21)	32 (5.13)	120 (11.68)	χ^2^ = 19.96***
Male	1,499 (90.79)	592 (94.87)	907 (88.32)	
Education				
No education or elementary undergraduate	376 (22.77)	145 (23.24)	231 (22.49)	χ^2^ = 0.95
Elementary graduate	284 (17.20)	107 (17.15)	177 (17.23)	
High school graduate	691 (41.85)	253 (40.54)	438 (42.65)	
College graduate or higher	300 (18.17)	119 (19.07)	181 (17.62)	
Employment				
Unemployed	79 (4.78)	39 (6.25)	40 (3.89)	χ^2^ = 11.98***
Employed	1,430 (86.61)	545 (87.34)	885 (86.17)	
Student	37 (2.24)	7 (1.12)	30 (2.92)	
Not in labor force	105 (6.36)	33 (5.29)	72 (7.01)	
Wealth tertile				χ^2^ = 3.78
Wealthiest tertile	549 (33.25)	334 (32.52)	215 (34.46)	
2nd wealthiest tertile	526 (31.86)	345 (33.59)	181 (29.01)	
Least wealthy tertile	576 (34.89)	348 (33.89)	228 (36.54)	
Nicotine dependence				
Smokes within 5 min of waking	239 (14.48)	122 (19.55)	117 (11.39)	χ^2^ = 71.82***
6 to 30 min	503 (30.47)	225 (36.06)	278 (27.07)	
31 to 60 min	229 (13.87)	87 (13.94)	142 (13.83)	
> 60 min	395 (23.92)	135 (21.63)	260 (25.32)	
Not daily smoker	285 (17.26)	55 (8.81)	230 (22.40)	
Type of Residence				
Rural	1,076 (65.17)	393 (62.98)	683 (66.50)	χ^2^ = 2.12
Urban	575 (34.83)	231 (37.02)	344 (33.50)	
Have at least 1 smoker in the household	1,610 (97.52)	616 (98.72)	994 (96.76)	χ^2^ = 5.98**
No smoker in the household	41 (2.48)	8 (1.29)	33 (3.21)	
Exposure to secondhand smoke	Mean = 1.29; SD = 1.01	Mean = 1.35; SD = 1.02	Mean = 1.25; SD = 1.00	t = -1.94*

***, **, * signify p-value levels of 1%, 5%, 10%, respectively. Means, standard deviations (SD), and t-tests were displayed for continuous variables while frequencies, column percentages, and χ^2^ tests were shown for categorical variables

We chose the two-class logit RE LCM since it yielded the lowest BIC and had the lowest proportion of predicted frequencies deviating from the observed frequencies to the total sample (see online S1 and S2 Tables for more information on the LCM).

**Fig 1:**
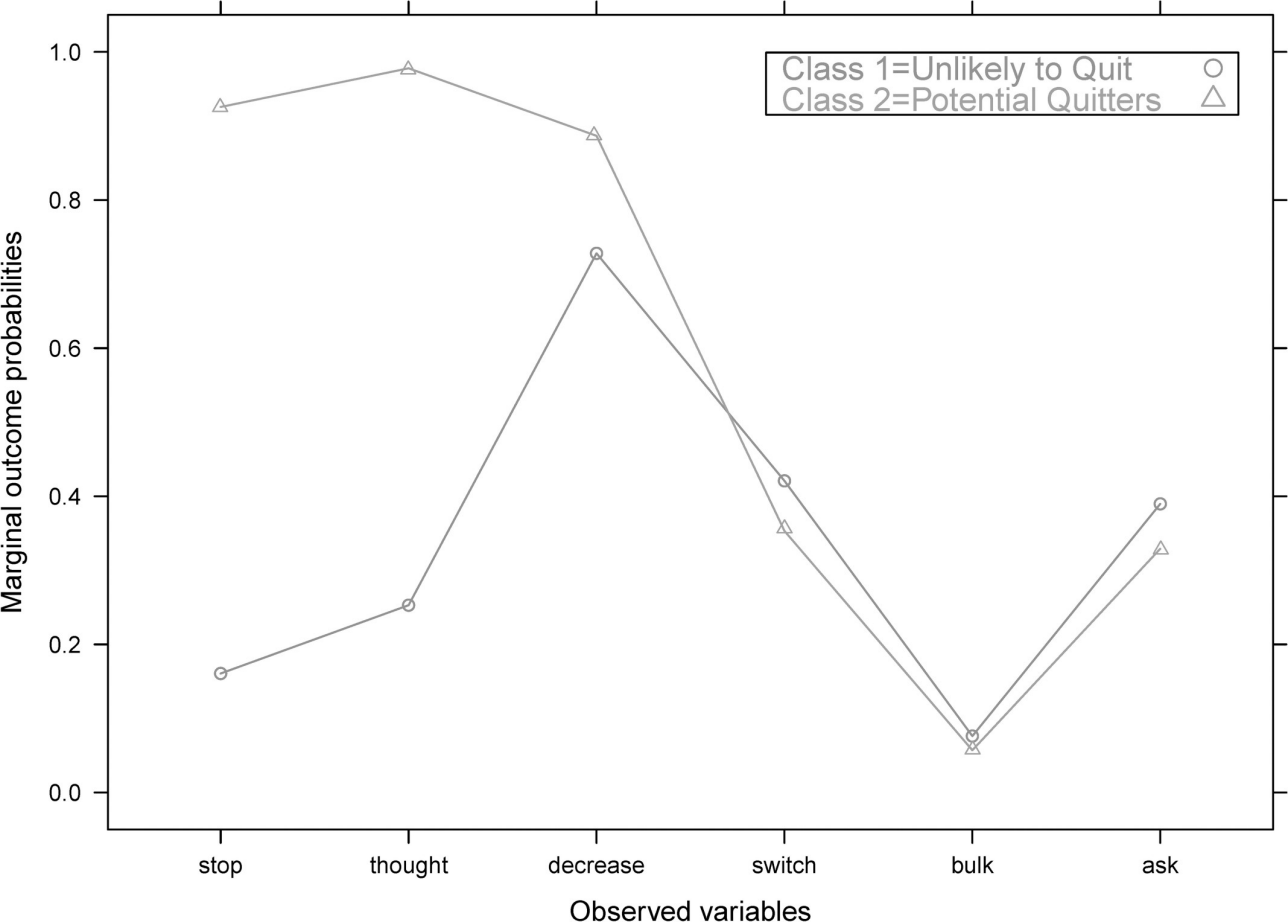
Marginal outcome probabilities by type of smoker.

We call classes 1 and 2 as “unlikely to quit” (UTQ) and “potential quitters” (PQ), respectively, because the two are clearly distinguished by their marginal outcome probability on *Stop*, *Thought*, and *Decrease–*the more drastic measures compared to the price-minimizing *Switch*, *Bulk*, and *Ask* tactics. Compared to those in the UTQ class, smokers classified under PQ have significantly higher marginal outcome probabilities for *Stop* and *Thought*, meaning, they are more likely to answer affirmatively to questions closely related to smoking cessation. PQs were also slightly more likely to *Decrease* than UTQ smokers. In terms of the marginal outcome probabilities of price-minimizing adaptations, those in the PQ and UTQ classes are almost similar (Fig 1). The UTQ individuals make up 37.38% (N = 624) of the sample while PQs make up 62.62% (N = 1,027). The UTQ smokers smoke three more sticks than PQs. As per the bivariate analyses, sex, employment status, nicotine dependence, presence of other smokers at home, and secondhand smoke exposure have statistical association with class membership (Table 2).

Table 3 reports the multivariate analyses results. The odds of females being in the UTQ group is about 51–59% less than males, while students have 55–63% lower odds of being in the UTQ class when compared to the employed. Adding nicotine dependence to the model slightly increased the odds of being in the UTQ group for females vs males and students vs employed. Those in the middle of the nicotine dependence scale have about 39–46% lower odds of being in the UTQ group versus the most-dependent group. Meanwhile, non-daily smokers or the least nicotine-dependent have 73% lower likelihood of being in the UTQ class than those who smoke within 5 minutes of waking (Columns 1 and 2).

**Table 3:** Regression results.

Independent Variables	Logistic regression (Odds Ratios)		Robust OLS	
	Belonging to the “unlikely to quit” class		Sticks smoked	
	[1]	[2]	[3]	[4]
Price			0.16	0.14
			(-0.04–0.36)	(-0.04–0.32)
Being “unlikely to quit” (“Potential quitters” as ref.)			2.93***	1.62***
			(2.13–3.72)	(0.89–2.34)
Age	1.00	1.00	0.06***	0.03**
	(0.99–1.01)	(0.99–1.01)	(0.03–0.09)	(0.01–0.06)
Female	0.41***	0.49***	-2.88***	-1.17**
	(0.27–0.63)	(0.32–0.76)	(-4.07 - -1.68)	(-2.19 - -0.16)
Education (College graduate or higher as ref.)				
No education or elementary undergraduate	0.92	0.91	-1.42*	-1.58**
	(0.65–1.32)	(0.63–1.30)	(-2.90–0.06)	(-2.89 - -0.27)
Elementary graduate	0.93	0.94	-0.85	-0.77
	(0.65–1.34)	(0.65–1.35)	(-2.26–0.56)	(-2.00–0.45)
High school graduate	0.84	0.82	-0.87	-1.06**
	(0.63–1.13)	(0.61–1.11)	(-2.06–0.33)	(-2.09 - -0.03)
Employment (Employed as ref.)				
Unemployed	1.49*	1.39	0.84	0.26
	(0.94–2.35)	(0.87–2.21)	(-0.83–2.51)	(-1.30–1.82)
Student	0.37**	0.45*	-3.21***	-2.01**
	(0.16–0.86)	(0.19–1.07)	(-5.17 - -1.25)	(-3.83 - -0.19)
Not in labor force	1.01	1.06	-2.06***	-1.53**
	(0.63–1.62)	(0.65–1.71)	(-3.42 - -0.70)	(-2.70 - -0.37)
Wealth tertile (Wealthiest tertile as reference)				
2nd	0.86	0.88	-0.26	-0.15
	(0.66–1.12)	(0.67–1.15)	(-1.24–0.72)	(-1.02–0.71)
1st	1.04	1.05	-0.94*	-0.77*
	(0.79–1.36)	(0.80–1.39)	(-1.96–0.07)	(-1.69–0.14)
Nicotine dependence (Smokes within 5 min of waking as ref.)				
6 to 30 min		0.79		-3.99***
		(0.58–1.09)		(-5.25 - -2.72)
31 to 60 min		0.61***		-4.02***
		(0.42–0.88)		(-5.57 - -2.47)
> 60 min		0.54***		-6.28***
		(0.38–0.75)		(-7.55 - -5.02)
Not daily smoker		0.27***		-12.46***
		(0.18–0.40)		(-13.62 - -11.30)
Rural (Urban as reference)	0.84	0.86	-0.36	-0.09
	(0.68–1.05)	(0.69–1.08)	(-1.18–0.46)	(-0.82–0.64)
Have at least 1 smoker in the household	2.16*	1.50	2.70**	-0.60
	(0.98–4.76)	(0.67–3.38)	(0.54–4.85)	(-2.29–1.09)
Exposure to secondhand smoke	1.08	1.04	0.33*	0.01
	(0.98–1.19)	(0.94–1.15)	(-0.06–0.72)	(-0.34–0.36)
Constant	0.35**	0.93	4.14***	14.40***
	(0.15–0.86)	(0.35–2.43)	(1.52–6.77)	(11.89–16.91)
Observations	1,651	1,651	1,651	1,651

95% confidence intervals in parentheses.

***, **, * signify p-value levels of 1%, 5%, 10%, respectively.

Robust OLS model results for both measures of smoking intensity is almost similar, so we only show coefficients for sticks smoked. Individuals in the UTQ class smoke about three more sticks than PQs but the difference declines to about 1.6 sticks when nicotine dependence is accounted for. Females smoke about three sticks less than males but the sex-gap in sticks smoked drops to 1.2 sticks when nicotine dependence is considered.

Compared to college graduates, those with less than elementary education smoke about 1.6 sticks less while high school graduates smoke about one stick less. Student smokers consume about 3 sticks less than the employed, although this figure drops to two sticks when nicotine dependence is added. Those who are not in the labor force smoke about two sticks less than the employed. Meanwhile, those in the lowest wealth tertile smoke about one stick less than those in the highest wealth tertile. Individuals who smoke within 6 to 30 min and within 31 to 60 min of waking are smoking four sticks less than those who smoke within 5 min of waking. Moreover, compared to the most nicotine-dependent group, those who smoke after an hour from waking consume six sticks less while the non-daily smokers consume 12 sticks less (Columns 3 and 4).

## Discussion

Since 2012, the Philippines made cigarettes significantly more expensive through a series of excise tax reforms. Only a few studies have been done to assess how smoking behavior was affected by the series of tax increases.

The tax could have stopped non-smokers from initiating smoking, made smokers quit, or reduced sticks smoked, but it could have also inadvertently pushed smokers to find ways to pay less to maintain current smoking levels. Our study tried to uncover the types of adaptations in response to the tax increase. Doing so may help understand why, despite the tax reform, average sticks smoked among smokers increased by almost one cigarette per day.

Two types of Filipino smokers emerged: one who made more drastic changes by thinking about quitting or attempting to quit, and another who did not drastically change smoking behavior and instead opted for price minimization. We called the groups “potential quitters (PQ)” and “unlikely to quit (UTQ),” respectively.

PQs had significantly higher probabilities of reporting that they tried to stop or thought about quitting smoking compared to UTQs. UTQs were less likely to resort to decreased consumption. The two classes did not differ much on the probability of price-minimization (i.e., switching to cheaper brands, buying in bulk, asking for cigarettes from others).

Adaptations varied by smokers’ sex, employment status, and nicotine dependence. However, we did not find any difference in class affinity on the basis of educational attainment or wealth, contrary to the existing body of evidence [33–36]. The absence of a significant link for educational attainment is likely due to small sample size and not necessarily the absence of any relationship. Related work done in Brazil [37] found that those with less than nine years of education are twice more likely to try quitting and switching to cheaper brands than those with nine or more years of schooling. In contrast, for a handful of developed western countries, the better educated had lower probabilities of having intentions to quit and adopting price-minimization strategies in response to a hypothetical cigarette price increase [38].

This study is an improvement of previous related work given that we considered possible adaptations as interrelated using LCM rather than looking at them in silos. One study [38] came up with non-mutually exclusive categories to derive a quit intention scale that runs from no change in behavior, adapting in ways other than quitting, adapting both other methods and quitting, and having quitting intentions alone albeit more arbitrarily than data driven. A handful of studies using the LCM to classify smokers in general exist but to the best of our knowledge, only one has been done specifically to classify smokers’ adaptive behavior towards price increases. Crespi et al. [39] uncovered five types of Italian smokers’ adaptations to price increases–typologies differed mainly by the level of sensitivity the smoker has towards risks and whether the smoker chose not to change habits through price-minimization tactics. Unlike our study, they were unable to observe quitting tendencies.

Our study has other limitations: (1) the presence of omitted variable bias despite best efforts to account for confounders, (2) the use of cross-sectional data which prevents us from testing whether our findings will hold over time, and (3) the use of self-reported behavior which can be tainted with social desirability bias.

Our study finds some evidence to suggest that the sin tax reform is associated with both smoking participation and smoking intensity because (1) 63% of smokers who were affected by the price increase considered stopping or quitting, and (2) these PQs smoked one to three sticks less than the UTQs. One strong hindering factor to potential cessation for the 37% in the UTQ class is nicotine dependence. Nicotine dependence significantly increased the odds of being in the UTQ class, consistent with previous studies [38, 40]. In sum, nicotine dependence hindered the impact of cigarette price increases not only on smoking cessation intentions but also smoking intensity.

We emphasize the need to take a comprehensive look at not only price but also non-price-based tobacco control interventions in the Philippines. First, that PQs are more likely to quit due price increases warrants a continuing monitoring and evaluation of the sin tax reform laws, notably, RA 11346 passed in 2019. The results of this paper builds on previous findings [40] by showing that smokers are not merely responding through independent decisions on cessation, demand reduction, or price minimization. Certain characteristics determine whether they are PQs or UTQs, and are therefore predisposed to respond in specific ways. Having a better understanding of the population of current smokers and those most likely to quit given tax-induced price increases can better guide policymaking.

Second, it is time to evaluate whether non-price-based interventions complement price-based measures. The Philippine Regulation Act of 2003 (RA 9211), passed almost two decades ago, provides the basis for most non-priced-based regulation, including smokefree places and prohibitions on advertisements and sale to minors. The law provides for the development of a National Smoking Cessation Program and creation of withdrawal clinics. In relation, the health department has instituted mandatory trainings on the policies and guidelines on smoking cessation since 2003 but only for government healthcare workers. Expanding its coverage should be seriously considered. Moreover, because nicotine dependence remains a strong predictor of adaptations to price increases, we suggest that the DOH provide services to address nicotine dependence. To the best of our knowledge, the closest program the DOH currently has for nicotine dependence is the National Quitline introduced in 2017, which had received only 983 calls [41]–a disproportionately small figure compared to the number of smokers in the country.

That the unlikely to quit smokers are less inclined to quit or reduce consumption points to the importance of complementing tax increases with targeted measures. Studies to better understand the UTQ group can help shed light on the best approach to address this group’s needs. Finally, research towards a better understanding of the smoking epidemic in the country is needed.

## Supporting information

S1 Table Bayesian information criterion for the latent class models. (DOCX)

S2 Table Frequency of response patterns, predicted values from the type of LCMs with lowest BICs, and the difference between actual frequency versus predicted frequency. (DOCX)

## References

[1] Murray C. J. L., Aravkin A. Y., Zheng P., Abbafati C., Abbas K. M., Abbasi-Kangevari M., et al. (2020). Global burden of 87 risk factors in 204 countries and territories, 1990–2019: a systematic analysis for the Global Burden of Disease Study 2019. *The Lancet*, 396(10258), 1223–1249. doi: 10.1016/S0140-6736(20)30752-2 33069327 PMC7566194

[2] Reitsma M. B., Fullman N., Ng M., Salama J. S., Abajobir A., Abate K. H., et al. (2017). Smoking prevalence and attributable disease burden in 195 countries and territories, 1990–2015: a systematic analysis from the Global Burden of Disease Study 2015. *The Lancet*, 389(10082), 1885–1906. doi: 10.1016/S0140-6736(17)30819-X 28390697 PMC5439023

[3] Department of Health—Philippines, & Philippine Statistics Authority. (2016). *Global Adult Tobacco Survey Philippines*: *Country Report 2015*. Retrieved from http://www.who.int/tobacco/surveillance/survey/gats/phl_country_report.pdf?ua=1

[4] World Bank. (2022). World Development Indicators. Retrieved May 1, 2022, from http://databank.worldbank.org

[5] Ng M., Freeman M. K., Fleming T. D., Robinson M., Dwyer-Lindgren L., Thomson B., et al. (2014). Smoking prevalence and cigarette consumption in 187 countries, 1980–2012. *JAMA—Journal of the American Medical Association*, 311(2), 183–192. doi: 10.1001/jama.2013.284692 24399557

[6] Costa M. L., Cohen J. E., Chaiton M. O., Ip D., McDonald P., & Ferrence R. (2010). “Hardcore”definitions and their application to a population-based sample of smokers. *Nicotine and Tobacco Research*, 12(8), 860–864. doi: 10.1093/ntr/ntq103 20601409

[7] Burns D. M., & Warner K. E. (2003). Smokers Who Have Not Quit: Is Cessation More Difficult and Should We Change Our Strategies? In *Those Who Continue to Smoke (Tobacco Control Monograph No*. *15)* (pp. 11–31). Bethesda, Maryland: National Cancer Institute.

[8] Congress of the Republic of the Philippines. (2012). *REPUBLIC ACT NO*. *10351; An Act Restructuring the Excise Tax on Alcohol and Tobacco Products by Amending Sections 141*, *142*, *143*, *144*, *145*, *8*, *131 and 288 of Republic Act No*. *8424*. *otherwise Known as the National Internal Revenue Code of 1997*, *as Amended by*. Metro Manila, Philippines.

[9] Cheng K. J. G., & Estrada M. A. G. (2020). Price elasticity of cigarette smoking demand in the Philippines after the 2012 Sin Tax Reform Act. *Preventive Medicine*, 134(December 2019), 106042. doi: 10.1016/j.ypmed.2020.106042 32097751

[10] Philippine Statistics Authority. (2017). *2015 Family Income and Expenditure Survey*. Quezon City, Philippines. Retrieved from https://psa.gov.ph/sites/default/files/FIES 2015 Final Report.pdf

[11] Philippine Statistics Authority. (2016). *Highlights on Household Population*, *Number of Households*, *and Average Household Size of the Philippines (2015 Census of Population)*. Quezon City, Philippines. Retrieved from https://psa.gov.ph/content/highlights-household-population-number-households-and-average-household-size-philippines

[12] Chaloupka F. J., Straif K., & Leon M. E. (2011). Effectiveness of tax and price policies in tobacco control. *Tobacco Control*, 20(3), 235–238. doi: 10.1136/tc.2010.039982 21115556

[13] International Agency for Research on Cancer. (2011). Effectiveness of tax and price policies for tobacco control: IARC Handbook of Cancer Prevention, Volume 14. *International Agency for Research on Cancer*, *WHO*, 14, 1–359. 10.1128/EC.4.12.2029

[14] Jha P., & Chaloupka F. (2000). The economics of global tobacco control. *BMJ*, 321, 358–361. doi: 10.1136/bmj.321.7257.358 10926598 PMC1118333

[15] Estrada M. A. G. (2018). *Smoking and Health in the Philippines*: *Five Years after Sin Tax Reform*. *CPBRD Policy Brief*. Retrieved from http://cpbrd.congress.gov.ph/images/PDF Attachments/CPBRD Policy Brief/PB2018-01_Smoking_and_Health_in_the_Philippines.pdf

[16] Centers for Disease Control and Prevention. (2022). Global Adult Tobacco Survey Comparison Fact Sheet: Indonesia 2011 and 2021. *Global Tobacco Surveillance System Data*. Retrieved September 11, 2022, from https://nccd.cdc.gov/GTSSDataSurveyResources/Ancillary/DownloadAttachment.aspx?ID=1017

[17] Centers for Disease Control and Prevention. (2012). Global Adult Tobacco Survey Fact Sheet: Malaysia 2011. *Global Tobacco Surveillance System Data*. Retrieved September 11, 2022, from https://nccd.cdc.gov/GTSSDataSurveyResources/Ancillary/DownloadAttachment.aspx?ID=1015%0A

[18] Centers for Disease Control and Prevention. (2012). Global Adult Tobacco Survey Comparison Fact Sheet: Thailand 2009 and 2011. *Global Tobacco Surveillance System Data*. Atlanta. Retrieved September 11, 2022, from https://nccd.cdc.gov/GTSSDataSurveyResources/Ancillary/DownloadAttachment.aspx?ID=1017%0A

[19] Centers for Disease Control and Prevention. (2010). Global Adult Tobacco Survey Fact Sheet: Viet Nam 2010. *Global Tobacco Surveillance System Data*. Retrieved September 11, 2022, from https://nccd.cdc.gov/GTSSDataSurveyResources/Ancillary/DownloadAttachment.aspx?ID=859

[20] Centers for Disease Control and Prevention. (2010). Global Adult Tobacco Survey Fact Sheet: Philippines 2009. *Global Tobacco Surveillance System Data*. Retrieved September 11, 2022, from https://nccd.cdc.gov/GTSSDataSurveyResources/Ancillary/DownloadAttachment.aspx?ID=70

[21] Rutstein S., & Johnson K. (2004). *The DHS Wealth Index*. Calverton, Maryland. Retrieved from https://dhsprogram.com/pubs/pdf/cr6/cr6.pdf

[22] Palipudi K. M., Gupta P. C., Sinha D. N., Andes L. J., Asma S., & McAfee T. (2012). Social Determinants of Health and Tobacco Use in Thirteen Low and Middle Income Countries: Evidence from Global Adult Tobacco Survey. *PLoS ONE*, 7(3), e33466. doi: 10.1371/journal.pone.0033466 22438937 PMC3306395

[23] Baker T., Piper M., McCarthy D., Bolt D., Smith S., Kim S. Y., et al. (2007). Time to first cigarette in the morning as an index of ability to quit smoking: Implications for nicotine dependence. *Nicotine and Tobacco Research*, 9(SUPPL. 4), 555–570. doi: 10.1080/14622200701673480 18067032 PMC2933747

[24] World Health Organization. (2010). *Economics of tobacco toolkit*: *economic analysis of demand using data from the Global Adult Tobacco Survey (GATS)*. Retrieved from http://whqlibdoc.who.int/publications/2010/9789241500166_eng.pdf

[25] Lazarsfeld P. F. (1950). The Logical and Mathematical Foundation of Latent Structure Analysis. In Stouffer S. A., Guttman L., Suchman E. A., Lazarsfeld P. F., Star S. A., & Clausen J. A. (Eds.), *tudies in Social Psychology in World War II*, *Vol*. *IV*, *Measurement and Prediction* (pp. 361–412). New Jersey: Princeton University Press.

[26] Vermunt J. K., & Magidson J. (2016). Latent Class Analysis. In Lewis-Beck M. S., Bryman A., & Liao T. F. (Eds.), *The Sage encyclopedia of social science research methods*. Sage Publications, Inc.

[27] Lazarsfeld P. F., & Henry N. W. (1968). *Latent Structure Analysis*. Boston, MA: Houghton Mifflin.

[28] Vacek P. M. (1985). The Effect of Conditional Dependence on the Evaluation of Diagnostic Tests. *Biometrics*, 41(4), 959. 10.2307/2530967 3830260

[29] Hagenaars J. A. (1988). Latent structure models with direct effects between indicators: local dependence models. *Sociological Methods and Research*, 16(3), 379–405. 10.1177/0049124188016003002

[30] Lindson N., Klemperer E., Hong B., Ordóñez-Mena J. M., & Aveyard P. (2019). Smoking reduction interventions for smoking cessation. *Cochrane Database of Systematic Reviews*, 2019(9). doi: 10.1002/14651858.CD013183.pub2 31565800 PMC6953262

[31] Beath K. J. (2017). randomLCA: An R Package for Latent Class with Random Effects Analysis. *Journal of Statistical Software*, 81(13). 10.18637/jss.v081.i13

[32] Qu Y., Tan M., & Kutner M. H. (1996). Random Effects Models in Latent Class Analysis for Evaluating Accuracy of Diagnostic Tests Author (s): Yinsheng Qu, Ming Tan and Michael H. Kutner Published by: International Biometric Society Stable URL: http://www.jstor.org/stable/2533043 Accessed. *Biometrics*, 52(3), 797–810. 8805757

[33] Cavelaars A. E. J. M., Kunst A. E., Mackenbach J. P., Geurts J. J. M., Crialesi R., Grötvedt L., et al. (2000). Educational differences in smoking: International comparison. *British Medical Journal*, 320(7242), 1102–1107. doi: 10.1136/bmj.320.7242.1102 10775217 PMC27351

[34] Hiscock R., Bauld L., Amos A., Fidler J. A., & Munafò M. (2012). Socioeconomic status and smoking: A review. *Annals of the New York Academy of Sciences*, 1248(1), 107–123. doi: 10.1111/j.1749-6632.2011.06202.x 22092035

[35] Pampel F. C., Krueger P. M., & Denney J. T. (2010). Socioeconomic disparities in health behaviors. *Annual Review of Sociology*, 36, 349–370. doi: 10.1146/annurev.soc.012809.102529 21909182 PMC3169799

[36] Pampel F. C., & Denney J. T. (2011). Cross-National Sources of Health Inequality: Education and Tobacco Use in the World Health Survey. *Demography*, 48(2), 653–674. doi: 10.1007/s13524-011-0027-2 21491184 PMC3296482

[37] Gigliotti A., Figueiredo V. C., Madruga C. S., Marques A. C. P. R., Pinsky I., Caetano R., et al. (2014). How smokers may react to cigarette taxes and price increases in Brazil: Data from a national survey. *BMC Public Health*, 14(1), 1–9. doi: 10.1186/1471-2458-14-327 24712903 PMC3991916

[38] Ross H., Blecher E., Yan L., & Cummings K. M. (2011). Predictors of what smokers say they will do in response to future price increases. Findings from the International Tobacco Control (ITC) Four Country Survey. *Nicotine & tobacco research*: *official journal of the Society for Research on Nicotine and Tobacco*, 13(6), 419–425. doi: 10.1093/ntr/ntr015 21385909 PMC3103716

[39] Crespi F., Liberati P., Paradiso M., Scialà A., & Tedeschi S. (2020). Smokers are different: The impact of price increases on smoking reduction and downtrading. *Economic Modelling*, (April). 10.1016/j.econmod.2020.04.004

[40] Cheng K. J. G., Lacaza R. M., & Estrada M. A. G. (2021). Varying Cost Minimization and Cessation Motivations in Response to Excise Tax Reform in the Philippines: A Cross-Sectional Study Using the 2015 Global Adult Tobacco Survey. *Asia-Pacific Journal of Public Health*. doi: 10.1177/10105395211065303 34894760

[41] Calica A., & Jaymalin M. (2019, July 31). DOH’s Quitline For Smokers Ready To Serve. *One News*. Retrieved from https://onenews.ph/doh-quitline-for-smokers-ready-to-serve.

