# Supplementary material for: A dichotomy of smokers in the Philippines following sin tax reform: Distinguishing potential quitters from those unlikely to quit
Source: PLoS One. 2022 Oct 13;17(10):e0275840. doi: 10.1371/journal.pone.0275840 (PMC9560617; doi:10.1371/journal.pone.0275840)
Supplement: S1 Table — (DOCX) [file pone.0275840.s001.docx]

**A dichotomy of smokers in the Philippines following sin tax reform: Distinguishing potential quitters from those unlikely to quit**

**SUPPLEMENTARY INFORMATION**

**S1 Table: Bayesian information criterion for the latent class models**

|  |  |  |  |
| --- | --- | --- | --- |
| **Number of classes** | **Standard LCM** | **Random effects LCM** | |
|  |  | **Logit scale** | **Probit scale** |
| 1 | 10,838.59 | 10,617.03 | 10,631.77 |
| 2 | 10,149.51 | 10,089.73 | 10,097.74 |
| 3 | 10,126.42 | 10,108.81 | 10,110.71 |
