# Supplementary material for: A dichotomy of smokers in the Philippines following sin tax reform: Distinguishing potential quitters from those unlikely to quit
Source: PLoS One. 2022 Oct 13;17(10):e0275840. doi: 10.1371/journal.pone.0275840 (PMC9560617; doi:10.1371/journal.pone.0275840)
Supplement: S2 Table — (DOCX) [file pone.0275840.s002.docx]

**A dichotomy of smokers in the Philippines following sin tax reform: Distinguishing potential quitters from those unlikely to quit**

**SUPPLEMENTARY INFORMATION**

**S2 Table: Frequency of response patterns, predicted values from the type of LCMs with lowest BICs, and the difference between actual frequency versus predicted frequency**

| Pattern of responses (Stop, Thought, Decrease, Switch, Bulk, Ask) | | | | | | Actual | Standard LCM | | RE LCM with Logit | | RE LCM with Probit | |
| --- | --- | --- | --- | --- | --- | --- | --- | --- | --- | --- | --- | --- |
|  |  |  |  |  |  | Freq | Predicted | vs Actual | Predicted | vs Actual | Predicted | vs Actual |
| 1 | 1 | 1 | 0 | 0 | 0 | 358 | 335.03 | 22.97 | 349.67 | 8.33 | 347.01 | 10.99 |
| 1 | 1 | 1 | 1 | 0 | 0 | 167 | 193.75 | 26.75 | 175.49 | 8.49 | 178.00 | 11.00 |
| 1 | 1 | 1 | 1 | 0 | 1 | 147 | 109.70 | 37.30 | 129.96 | 17.04 | 126.29 | 20.71 |
| 1 | 1 | 1 | 0 | 0 | 1 | 145 | 172.50 | 27.50 | 155.18 | 10.18 | 158.01 | 13.01 |
| 0 | 0 | 1 | 0 | 0 | 0 | 116 | 111.75 | 4.25 | 105.26 | 10.74 | 105.27 | 10.73 |
| 0 | 0 | 0 | 0 | 0 | 0 | 60 | 62.45 | 2.45 | 65.05 | 5.05 | 63.09 | 3.09 |
| 0 | 0 | 1 | 1 | 0 | 1 | 60 | 56.21 | 3.79 | 42.64 | 17.36 | 41.59 | 18.41 |
| 1 | 1 | 0 | 0 | 0 | 0 | 57 | 40.61 | 16.39 | 57.81 | 0.81 | 57.52 | 0.52 |
| 0 | 1 | 1 | 0 | 0 | 0 | 55 | 54.11 | 0.89 | 58.78 | 3.78 | 58.50 | 3.50 |
| 0 | 0 | 1 | 1 | 0 | 0 | 54 | 49.18 | 4.82 | 58.30 | 4.30 | 58.74 | 4.74 |
| 0 | 1 | 1 | 1 | 0 | 0 | 38 | 32.61 | 5.39 | 31.04 | 6.96 | 31.14 | 6.86 |
| 0 | 0 | 1 | 0 | 0 | 1 | 36 | 40.02 | 4.02 | 50.28 | 14.28 | 50.85 | 14.85 |
| 0 | 1 | 1 | 1 | 0 | 1 | 33 | 40.35 | 7.35 | 27.29 | 5.71 | 25.78 | 7.22 |
| 1 | 0 | 1 | 0 | 0 | 0 | 25 | 21.81 | 3.19 | 22.16 | 2.84 | 22.21 | 2.79 |
| 0 | 0 | 0 | 0 | 0 | 1 | 24 | 22.35 | 1.65 | 20.09 | 3.91 | 20.49 | 3.51 |
| 0 | 1 | 0 | 0 | 0 | 0 | 23 | 19.71 | 3.29 | 18.97 | 4.03 | 19.20 | 3.80 |
| 0 | 1 | 1 | 0 | 0 | 1 | 20 | 23.15 | 3.15 | 27.00 | 7.00 | 27.17 | 7.17 |
| 1 | 0 | 1 | 1 | 0 | 1 | 18 | 19.68 | 1.68 | 13.30 | 4.70 | 12.62 | 5.38 |
| 1 | 1 | 0 | 0 | 0 | 1 | 17 | 20.53 | 3.53 | 15.96 | 1.04 | 16.26 | 0.74 |
| 1 | 1 | 1 | 0 | 1 | 0 | 17 | 23.14 | 6.14 | 15.79 | 1.21 | 15.60 | 1.40 |
| 0 | 0 | 0 | 1 | 0 | 0 | 16 | 19.83 | 3.83 | 23.30 | 7.30 | 23.75 | 7.75 |
| 1 | 1 | 1 | 1 | 1 | 0 | 14 | 13.17 | 0.83 | 13.23 | 0.77 | 13.92 | 0.08 |
| 1 | 1 | 1 | 1 | 1 | 1 | 12 | 6.95 | 5.05 | 16.61 | 4.61 | 16.71 | 4.71 |
| 1 | 1 | 0 | 1 | 0 | 0 | 11 | 22.44 | 11.44 | 18.06 | 7.06 | 18.40 | 7.40 |
| 1 | 0 | 1 | 0 | 0 | 1 | 9 | 8.08 | 0.92 | 11.93 | 2.93 | 11.91 | 2.91 |
| 1 | 1 | 0 | 1 | 0 | 1 | 9 | 11.48 | 2.48 | 8.54 | 0.46 | 8.25 | 0.75 |
| 1 | 1 | 1 | 0 | 1 | 1 | 9 | 11.90 | 2.90 | 11.69 | 2.69 | 12.36 | 3.36 |
| 0 | 0 | 0 | 1 | 0 | 1 | 8 | 7.17 | 0.83 | 11.21 | 3.21 | 11.49 | 3.49 |
| 1 | 0 | 0 | 0 | 0 | 0 | 8 | 11.44 | 3.44 | 7.65 | 0.35 | 7.73 | 0.27 |
| 0 | 0 | 0 | 0 | 1 | 1 | 7 | 1.86 | 5.14 | 1.01 | 5.99 | 1.03 | 5.97 |
| 0 | 1 | 0 | 1 | 0 | 0 | 7 | 6.95 | 0.05 | 7.14 | 0.14 | 7.54 | 0.54 |
| 1 | 0 | 1 | 1 | 0 | 0 | 7 | 12.68 | 5.68 | 13.76 | 6.76 | 13.69 | 6.69 |
| 0 | 0 | 0 | 0 | 1 | 0 | 6 | 5.20 | 0.80 | 2.09 | 3.91 | 1.89 | 4.11 |
| 0 | 1 | 0 | 0 | 0 | 1 | 6 | 7.48 | 1.48 | 6.20 | 0.20 | 6.55 | 0.55 |
| 0 | 0 | 1 | 0 | 1 | 0 | 5 | 9.30 | 4.30 | 5.23 | 0.23 | 5.29 | 0.29 |
| 1 | 1 | 0 | 0 | 1 | 0 | 5 | 2.84 | 2.16 | 1.62 | 3.38 | 1.38 | 3.62 |
| 1 | 0 | 0 | 0 | 0 | 1 | 4 | 4.12 | 0.12 | 2.89 | 1.11 | 2.99 | 1.01 |
| 1 | 0 | 0 | 1 | 0 | 1 | 4 | 1.37 | 2.63 | 2.24 | 1.76 | 2.26 | 1.74 |
| 0 | 0 | 0 | 1 | 1 | 0 | 3 | 1.65 | 1.35 | 1.17 | 1.83 | 1.19 | 1.81 |
| 0 | 0 | 1 | 1 | 1 | 0 | 3 | 3.17 | 0.17 | 4.44 | 1.44 | 4.74 | 1.74 |
| 0 | 1 | 1 | 0 | 1 | 0 | 3 | 4.16 | 1.16 | 2.79 | 0.21 | 2.83 | 0.17 |
| 0 | 1 | 1 | 0 | 1 | 1 | 3 | 1.75 | 1.25 | 2.45 | 0.55 | 2.64 | 0.36 |
| 0 | 1 | 1 | 1 | 1 | 0 | 3 | 1.89 | 1.11 | 2.83 | 0.17 | 3.03 | 0.03 |
| 1 | 0 | 0 | 1 | 0 | 0 | 3 | 3.69 | 0.69 | 3.34 | 0.34 | 3.45 | 0.45 |
| 0 | 0 | 1 | 0 | 1 | 1 | 2 | 3.33 | 1.33 | 3.83 | 1.83 | 4.11 | 2.11 |
| 0 | 1 | 0 | 1 | 1 | 0 | 2 | 0.56 | 1.44 | 0.46 | 1.54 | 0.50 | 1.50 |
| 0 | 1 | 1 | 1 | 1 | 1 | 2 | 1.25 | 0.75 | 4.23 | 2.23 | 4.29 | 2.29 |
| 1 | 0 | 1 | 0 | 1 | 0 | 2 | 1.79 | 0.21 | 1.24 | 0.76 | 1.29 | 0.71 |
| 1 | 0 | 1 | 0 | 1 | 1 | 2 | 0.66 | 1.34 | 1.19 | 0.81 | 1.31 | 0.69 |
| 1 | 1 | 0 | 0 | 1 | 1 | 2 | 1.43 | 0.57 | 0.77 | 1.23 | 0.72 | 1.28 |
| 1 | 0 | 0 | 1 | 1 | 0 | 1 | 0.30 | 0.70 | 0.23 | 0.77 | 0.25 | 0.75 |
| 1 | 0 | 1 | 1 | 1 | 1 | 1 | 0.49 | 0.51 | 2.16 | 1.16 | 2.25 | 1.25 |
| 1 | 1 | 0 | 1 | 1 | 0 | 1 | 1.56 | 0.56 | 0.87 | 0.13 | 0.81 | 0.19 |
| 1 | 1 | 0 | 1 | 1 | 1 | 1 | 0.79 | 0.21 | 0.73 | 0.27 | 0.65 | 0.35 |
| Sum | | | | | | 1,651 |  | 253.93 |  | 205.87 |  | 221.32 |
| Percent to total sample ^a^ | | | | | |  |  | 15.38% |  | 12.47% |  | 13.41% |

Notes: In the pattern of responses columns, 1=yes, 0=no. The order of responses are arranged by: Stop, Thought, Decrease, Switch, Bulk, Ask so a 111000 means that the respondent said yes to Stop, Quit, and Decrease but no to the other questions. The “vs Actual” column shows the absolute difference between the actual frequency and the predicted frequency. ^a^ This is the sum of absolute differences divided by the total sample size.
